# Supplementary material for: Assessment of the Microbial Spoilage and Quality of Marinated Chicken Souvlaki through Spectroscopic and Biomimetic Sensors and Data Fusion
Source: Microorganisms. 2022 Nov 14;10(11):2251. doi: 10.3390/microorganisms10112251 (PMC9698993; doi:10.3390/microorganisms10112251)
Supplement: Supplementary file 1 [file microorganisms-10-02251-s001.zip › microorganisms-2037554-supplementary.pdf]

Table S1: SVM-R optimized parameters and kernel function combinations (for each sensor model) indicating the minimum MSE.

	Box of constraction, c	epsilon, e	Kernel function
**FT-IR**	2.5095	0.00566	Linear
**MSI**	1.2616	0.0023	Linear
**E-nose**	0.0976	0.3806	Linear
**FT-IR/MSI**	16.322	0.2407	Linear
**E-nose/FT-IR**	0.0157	0.0342	Linear
**MSI/E-nose**	216.512	0.0031	Linear
**3-sensors**	0.0289	0.0315	Linear
